# A Bidirectional Mendelian Randomization Study of Selenium Levels and Ischemic Stroke

**DOI:** 10.3389/fgene.2022.782691

**Published:** 2022-04-13

**Authors:** Hui Fang, Weishi Liu, Luyang Zhang, Lulu Pei, Yuan Gao, Lu Zhao, Rui Zhang, Jing Yang, Bo Song, Yuming Xu

**Affiliations:** Department of Neurology, The First Affiliated Hospital of Zhengzhou University, Zhengzhou, China

**Keywords:** selenium, stroke, trace element, cause, Mendelian randomization (MR)

## Abstract

**Background:** Previous observational studies have shown that circulating selenium levels are inversely associated with ischemic stroke (IS). Our aims were to evaluate the causal links between selenium levels and IS, and its subtypes by Mendelian randomization (MR) analysis.

**Methods:** We used the two-sample Mendelian randomization (MR) method to determine whether the circulating selenium levels are causally associated with the risk of stroke. We extracted the genetic variants (SNPs) associated with blood and toenail selenium levels from a large genome-wide association study (GWAS) meta-analysis. Inverse variance-weighted (IVW) method was used as the determinant of the causal effects of exposures on outcomes.

**Results:** A total of 4 SNPs (rs921943, rs6859667, rs6586282, and rs1789953) significantly associated with selenium levels were obtained. The results indicated no causal effects of selenium levels on ischemic stroke by MR analysis (OR = 0.968, 95% CI 0.914–1.026, p = 0.269). Meanwhile, there was no evidence of a causal link between circulating selenium levels and subtypes of IS.

**Conclusion:** The MR study indicated no evidence to support the causal links between genetically predicted selenium levels and IS. Our results also did not support the use of selenium supplementation for IS prevention at the genetic level.

## Introduction

Ischemic stroke (IS) is one of the leading causes of death worldwide and a major cause of serious long-term disability (Campbell et al., 2019). Although IS mortality has been declining globally over the past 2 decades, the number of IS incidents, IS survivors, IS-related deaths, and overall disability-adjusted life years (DALY) lost remains significant and increases year by year (Krishnamurthi et al., 2013). Therefore, early identification of the subjects with a high risk of developing or relapsing IS is of great importance. In addition, the benefit of effective medication for IS (i.e., alteplase) is time-dependent, which limits the wide application of alteplase practice (Phipps and Cronin, 2020). The major challenge of developing new anti-stroke drugs is the presence of the blood–brain barrier and blood circulation gaps, as well as the complexity of signal transduction processes and inflammatory response (Amani et al., 2017; Saxton and Sabatini, 2017). Moreover, fast metabolization clearance from blood circulation and poor transport across the blood–brain barrier hinder the efficacy of most central venous system medications (Amani et al., 2017; Amani et al., 2019). All in all, further investigation of risk factors of IS and targeted therapy strategies is warranted.

The major modifiable risk factors of IS include hypertension, diabetes mellitus, hyperlipidemia, and smoking (Go et al., 2014; Feigin et al., 2016). In addition, some trace elements, particularly essential trace elements, have been reported to be associated with IS (Zecca et al., 2004; Scheiber et al., 2014). Selenium is one of the essential trace elements involved in human physiological processes, metabolism, antioxidant defense, immune regulation, and so on (Burk et al., 2014). The main functions of selenoproteins, the main functional form of selenium, in the neural cells are modulation of neurogenesis, regulation of Ca^2+^ channels, and maintenance of the redox balance (Cardoso et al., 2015). Reported *in vitro* studies show that selenium protects mitochondrial functional performance, stimulates mitochondrial biogenesis, and reduces infarct volume after focal cerebral ischemia, through an autophagy-dependent mechanism (Mehta et al., 2012).

**FIGURE 1:**
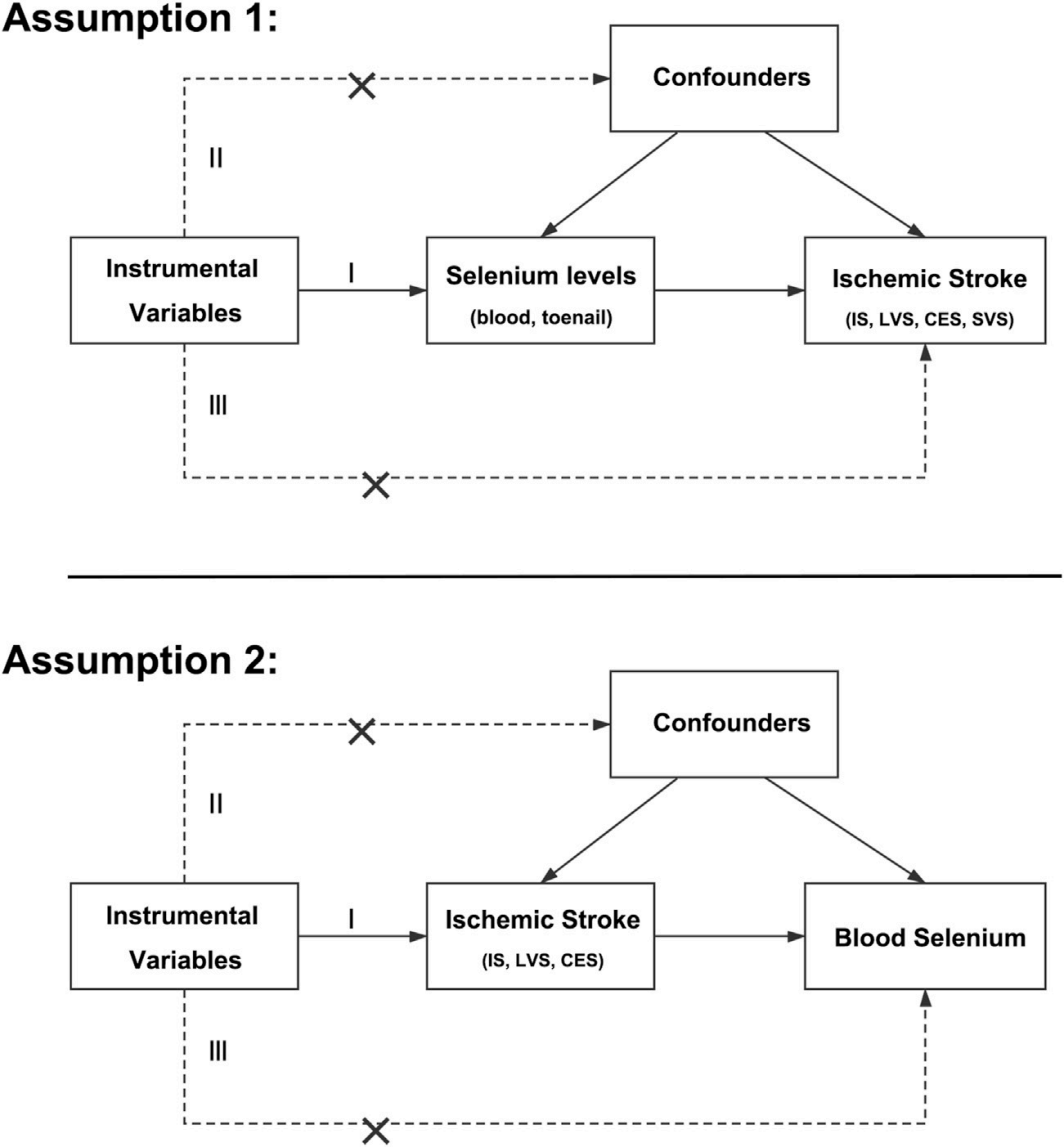
Main assumptions of the Mendelian randomization study of selenium levels and ischemic stroke. IS, ischemic stroke; LVS, large-vessel atherosclerosis stroke; CES, cardio-embolic stroke; SVS, small-vessel occlusion stroke.

Evidence from observational studies indicated that circulating selenium levels were inversely correlated with certain cardiovascular outcomes with a possible U-shaped association, and beneficial effects against IS were found in IS patients as well (Flores-Mateo et al., 2006; Stranges et al., 2010; Rees et al., 2013). However, results from clinical trials were controversial. Specifically, reports of the Selenium and Vitamin E Cancer Prevention Trial (SELECT) and Nutritional Prevention of Cancer Trial (NPC) found no beneficial effects on the incidence and mortality of coronary heart disease and stroke (Stranges et al., 2006; Lippman et al., 2009). In addition, results from a population-based survey revealed that blood selenium concentration might be inversely associated with the prevalence of stroke, and the relationship was non-linear (Hu et al., 2019). However, due to selection bias and reverse causation, the association between selenium levels and the risk of IS may be overestimated. In addition, whether selenium had different impacts on IS subtypes remains unclear. Mendelian randomization (MR), which uses genetic variants as instrumental variables, is a powerful method for inferring causal links between exposures and outcomes. MR analysis uses genetic variants associated with the selenium levels, as the random allocation in randomized controlled trials, to determine the causal effect of the selenium levels on IS, and vice versa (Davies et al., 2018). Since the genes are randomly allocated at conception, genetically predicted selenium levels are not associated with any potential confounders. In addition, random allocation at birth can also avoid the bias caused by reverse causation, as other factors, like disease status cannot affect the genes (Davies et al., 2018). MR analysis was established by three main assumptions (Emdin et al., 2017). First, instrumental variables were significantly associated with the exposure. Next, no links between instrumental variables and confounders were identified. Last, the impact of instrumental variables on outcome was only via exposure (Figure 1). Therefore, MR analysis could overcome the limitations of observational studies and provide insights into the association between selenium and IS. And our aims were to evaluate the causal links between selenium levels and IS and their subtypes by MR analysis.

## Materials and Methods

### Data Sources

The genetic variants associated with selenium levels were obtained from a large genome-wide association study (GWAS) meta-analysis of blood selenium (*n* = 5,477) and toenail selenium (*n* = 4,162) levels in people of European ancestry (Evans et al., 2013; Cornelis et al., 2015). The genetic variants associated with IS were obtained from a large GWAS by the MEGASTROKE consortium with 34,217 cases and 406,111 controls (Malik et al., 2018). Based on the Trial of ORG 10172 in Acute Stroke Treatment (TOAST) classification, all IS cases could be further divided into large-vessel atherosclerosis stroke (LVS, *n* = 4,373), cardio-embolic stroke (CES, *n* = 7,193), and small-vessel occlusion stroke (SVS, *n* = 5,386) (Adams et al., 1993; Malik et al., 2018). To perform bidirectional MR analysis, the GWAS of the blood selenium level was used as the outcome dataset (Evans et al., 2013).

Sample overlap was calculated in percentages by dividing the number of participants in the GWAS of selenium levels by the number of participants in the respective cohorts in the GWAS of IS and its subtypes (Evans et al., 2013; Cornelis et al., 2015; Malik et al., 2018). An acceptable level of population overlaps between selenium and IS and its subtypes GWAS datasets was 0.22–0.63%.

### Selection Criteria of Genetic Variants

We selected genetic variants associated with selenium levels, IS of all causes, LAS, CES, and SVS at genome-wide significance (*p* < 5 × 10^−8^) as instrumental variables. Then linkage disequilibrium was tested among the preliminarily selected single-nucleotide polymorphisms (SNPs), and those with *r* ^2^ > 0.01 in the 1000 Genome Project of Europeans were excluded. The proportion of variance (*R* ^2^) in the selenium levels explained by the selected genetic variants was calculated using the following formula: *R* ^2^ = 2 × *β* ^2^ × (1-EAF) × EAF, where *β* represents the estimated effect of the genetic variant and EAF represents the effect allele frequency (Palmer et al., 2012). In addition, *F*-statistic was calculated using the following formula: *F* = *R* ^2^ × (*N*-*k*-1)/*k* (1-*R* ^2^), where *R* ^2^ represents the proportion of variance explained by the genetic variants, *N* represents the sample size, and *k* represents the number of included SNPs (Palmer et al., 2012). The SNPs with an *F*-statistic <10 were considered weak instruments and were excluded from the MR analysis (Burgess et al., 2011).

Then, the corresponding genetic variants were obtained from the dataset of outcomes (IS or selenium). If selenium-associated SNPs were not available in the outcome datasets, then a proxy SNP in linkage disequilibrium (*r* ^2^ > 0.9) was searched online (https://ldlink.nci.nih.gov/) as replacement and used in the further analysis.

All genetic variants were searched in the PhenoScanner V2 database to assess whether those variants were significantly associated with the risk factors for IS and its subtypes (Kamat et al., 2019).

### Statistical Analysis

All analyses were conducted by R software (version 4.0.3) with R packages TwoSampleMR, MRPRESSO, and MendelianRandomization (Yavorska and Burgess, 2017; Hemani et al., 2018; Verbanck et al., 2018). The estimated effect for blood and toenail selenium levels was presented as *Z*-score units per effect allele (Evans et al., 2013; Cornelis et al., 2015). Therefore, the *Z*-score was converted to *β* and standard error values by the formulas described previously (Kho et al., 2019). The inverse variance-weighted (IVW) method was used as the determinants of the causal effects of exposures on outcomes (Hemani et al., 2018). We also performed MR-Egger, simple median, weighted median, simple mode, weighted mode, robust adjusted profile score (RAPS), Bayesian weighted Mendelian randomization (BWMR), Mendelian randomization pleiotropy residual sum and outlier (MR-PRESSO), and Mendelian randomization least absolute shrinkage and selection operator (MR-LASSO) methods (Bowden et al., 2015; Bowden et al., 2016; Hartwig et al., 2017; Verbanck et al., 2018; Zhao et al., 2020). Sensitivity tests including the heterogeneity test (Cochrane’s *Q* test), pleiotropy test (MR-Egger intercept test), and leave-one-out test were performed (Bowden et al., 2015). Bonferroni correction (corrected *p* = 0.05/*X*/*Y*, where *X* represents the number of exposures and *Y* represents the number of outcomes) was used for multiple comparisons.

### Power Calculation for Bidirectional Mendelian Randomization Analyses

Statistical power for the bidirectional MR analyses was calculated by mRnd (Brion et al., 2013). The minimum effect estimates of selenium levels required to achieve a power of 80% based on the sample size of the outcome datasets and the R2 by the IVs were calculated and is given in Supplementary Table S1.

## Results

### The Causal Effects of Selenium Levels on Ischemic Stroke

**FIGURE 2:**
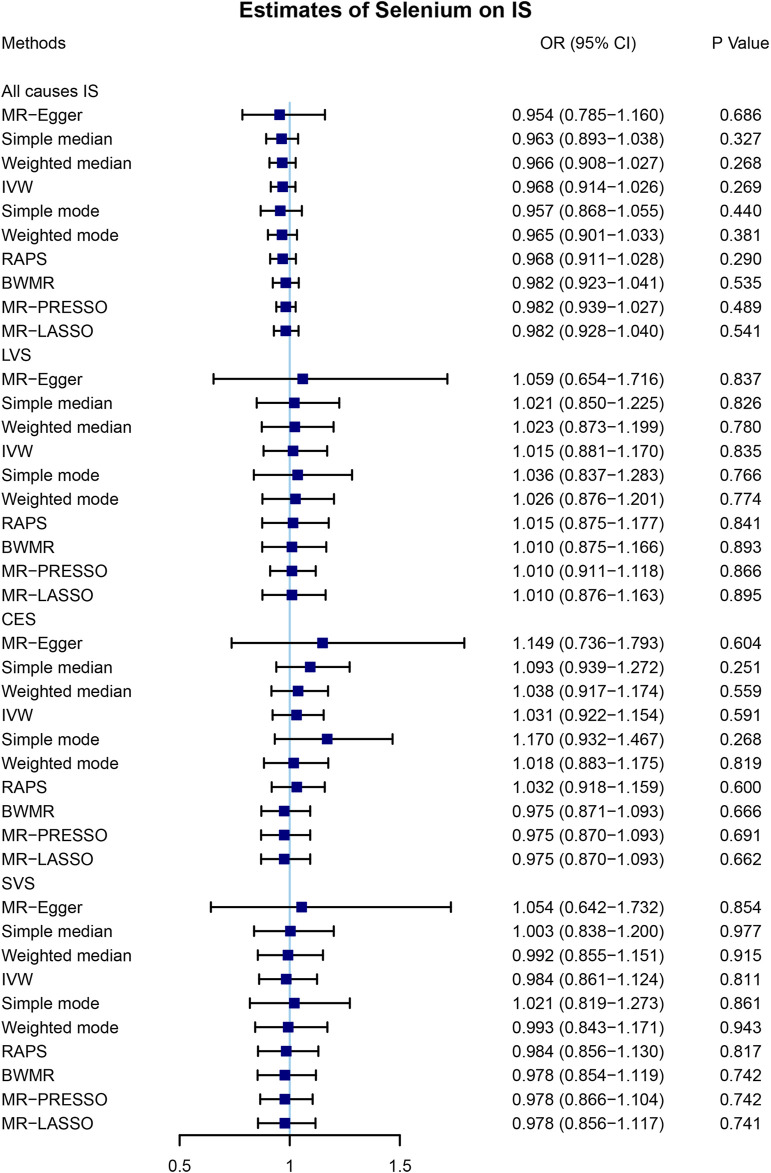
Mendelian randomization analysis of the causal effects of selenium levels on ischemic stroke. A total of 4 SNPs significantly associated with selenium levels were obtained. MR, Mendelian randomization; IS, ischemic stroke; SNP, single-nucleotide polymorphism; OR, odds ratio; CI, confidential interval; IVW, inverse variance-weighted; RAPS, robust adjusted profile score; BWMR, Bayesian weighted Mendelian randomization; MR-PRESSO, Mendelian randomization pleiotropy residual sum and outlier; MR-LASSO, Mendelian randomization least absolute shrinkage and selection operator; LVS, large-vessel atherosclerosis stroke; CES, cardio-embolic stroke; SVS, small-vessel occlusion stroke.

A total of 4 SNPs (rs921943, rs6859667, rs6586282, and rs1789953) significantly associated with selenium levels were obtained (Table 1). The 4 SNPs explained 5.9% of the variance in the selenium levels, and the corresponding *F*-statistic was about 151.8. Then, we used PhenoScanner V2 to find whether horizontal pleiotropy existed in the 4 SNPs (Kamat et al., 2019). We found that rs6586282 was significantly associated with plasma homocysteine levels, and rs921943 was associated with height. In MR analysis, the IVW method indicated no causal effects of selenium levels on IS of all causes (OR = 0.968, 95% CI 0.914–1.026, *p* = 0.269), LVS (OR = 1.015, 95% CI 0.881–1.170, *p* = 0.835), CES (OR = 1.031, 95% CI 0.922–1.154, *p* = 0.591), and SVS (OR = 0.984, 95% CI 0.861–1.124, *p* = 0.811) (Supplementary Table S2 and Figure 2). Heterogeneity tests indicated no heterogeneities of the genetic variants for IS of all causes (*p* = 0.626), LVS (*p* = 0.472), CES (*p* = 0.259), and SVS (*p* = 0.293) (Supplementary Table S3), and pleiotropy tests indicated no pleiotropy of the genetic variants for IS of all causes (*p* = 0.896), LVS (*p* = 0.874), CES (*p* = 0.669), and SVS (*p* = 0.802) (Supplementary Table S3). Leave-one-out analysis indicated that the results were still powerful and stable even if they excluded any single SNP (Supplementary Figure S1). Likewise, excluding the effect of rs6586282 did not significantly change the results of MR analysis (Supplementary Figure S1). Altogether, our results indicated no causal effects of selenium levels on IS and its subtypes by MR analysis.

**TABLE 1:** SNPs significantly associated with selenium levels and included in the MR study.

SNP	Nearby gene	Ch	E/O allele	EAF	*N*	β	SE	*Z*-score	*p*-value	*R* ^2^
rs921943	DMGDH	5	T/C	0.29	9,639	0.295	0.022	13.14	1.90 × 10^−39^	0.0358
rs6859667	HOMER1	5	T/C	0.96	9,639	−0.360	0.052	−6.92	4.40 × 10^−12^	0.0099
rs6586282	CBS	21	T/C	0.17	9,639	−0.160	0.027	−5.89	3.96 × 10^−9^	0.0072
rs1789953	CBS	21	T/C	0.14	9,639	0.162	0.029	5.52	3.40 × 10^−8^	0.0063

SNP, single-nucleotide polymorphism; MR, Mendelian randomization; Ch, chromosome; SE, standardized error; E/O, effect or other; EAF, effect allele frequency.

### The Causal Effects of Ischemic Stroke on Blood Selenium

**FIGURE 3:**
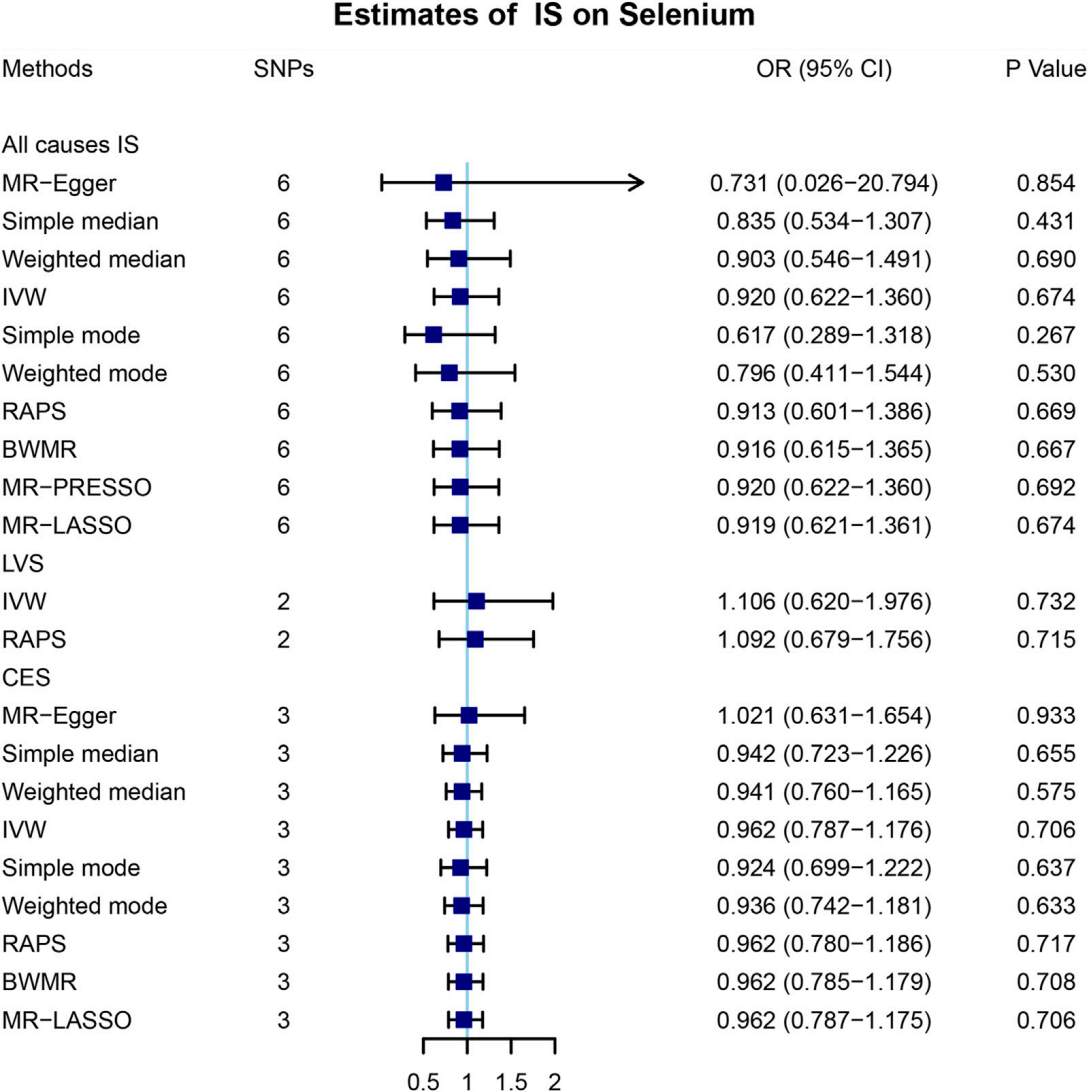
Mendelian randomization analysis of the causal effects of ischemic stroke on blood selenium levels. A total of 6, 2, and 3 SNPs significantly associated with IS of all causes, LVS, and CES were obtained in the reverse Mendelian randomization analysis. MR, Mendelian randomization; IS, ischemic stroke; SNP, single-nucleotide polymorphism; OR, odds ratio; CI, confidential interval; IVW, inverse variance-weighted; RAPS, robust adjusted profile score; BWMR, Bayesian weighted Mendelian randomization; MR-PRESSO, Mendelian randomization pleiotropy residual sum and outlier; MR-LASSO, Mendelian randomization least absolute shrinkage and selection operator; LVS, large-vessel atherosclerosis stroke; CES, cardio-embolic stroke; SVS, small-vessel occlusion stroke.

To further explore the association between the blood selenium level and IS and its subtypes, we further performed bidirectional MR analysis to estimate the causal effects of IS and its subtypes on blood selenium level. Overall, 9, 4, and 4 SNPs significantly associated with IS of all causes, LVS, and CES were obtained, respectively (Supplementary Table S2). No SNPs significantly associated with SVS were identified. After testing for linkage disequilibrium, 7, 2, and 3 SNPs significantly associated with IS of all causes, LVS, and CES remained, respectively (Table 2; Supplementary Tables S2–S4). By using the IVW method, our results indicated no causal effects of IS of all causes (OR = 0.920, 95% CI 0.622–1.360, *p* = 0.674), LVS (OR = 1.105, 95% CI 0.620–1.976, *p* = 0.732), and CES (OR = 0.962, 95% CI 0.787–1.176, *p* = 0.706) on the blood selenium level (Supplementary Tables S2–S4 and Figure 3). Sensitivity analysis indicated heterogeneities in the analysis of LVS (*p* = 0.027) and blood selenium level (Table 3). No heterogeneities were identified in the analysis of IS of all cause (*p* = 0.352) or CES (*p* = 0.692) (Table 3). The pleiotropy test indicated no pleiotropy (IS of all causes: *p* = 0.404; CES: *p* = 0.672) among the genetic variants (Table 3). Leave-one-out analysis indicated that the results of our analysis were powerful (Supplementary Figure S2). Altogether, our results indicated no causal effects of IS and its subtypes on the blood selenium level by MR analysis.

**TABLE 2:** MR results of the effect of IS and its subtypes on selenium levels.

SNP	Nearby Gene	Ch.	E/O Allele	EAF	*N*	Exposure			Outcome^a^				
						β	SE	*p*	β		SE		*p*
IS of all causes													
rs2758612^b^	PMF1-BGLAP	1	T/C	0.645	440,328	0.065	0.011	3.68 × 10^−9^	NA	NA		NA	
rs34311906^b^	ANK2	4	C/T	0.402	440,328	0.065	0.011	1.07 × 10^−8^	NA	NA		NA	
rs2634074^b^	RP11-119H12.3	4	T/A	0.212	440,328	0.094	0.012	5.90 × 10^−15^	0.018	0.037		0.620	
rs2066864	FGG	4	A/G	0.245	440,328	0.063	0.012	3.51 × 10^−8^	0.036	0.034		0.296	
rs11242678	RP11-157J24.2	6	T/C	0.255	440,328	0.072	0.011	2.70 × 10^−10^	0.031	0.034		0.358	
rs2107595	HDAC9	7	A/G	0.167	440,328	0.088	0.013	2.33 × 10^−11^	−0.034	0.041		0.412	
rs473238	WTAPP1	11	T/C	0.133	440,328	0.083	0.015	1.65 × 10^−8^	0.057	0.046		0.215	
rs3184504	SH2B3	12	T/C	0.472	440,328	0.078	0.010	1.23 × 10^−14^	−0.002	0.029		0.957	
rs4942561	LRCH1	13	T/G	0.759	440,328	0.066	0.012	1.77 × 10^−8^	−0.039	0.033		0.247	
LVS													
rs7610618^b^	SIAH2	3	T/C	0.013	150,765	0.845	0.149	1.44 × 10^−8^	NA	NA		NA	
rs2107595	HDAC9	7	A/G	0.168	150,765	0.236	0.032	1.44 × 10^−13^	−0.034	0.041		0.412	
rs10820405	LINC01492	9	G/A	0.815	150,765	0.181	0.033	4.51 × 10^−8^	−0.083	0.038		0.027	
rs476762^b^	MMP3	11	A/T	0.133	150,765	0.201	0.035	1.22 × 10^−8^	−0.056	0.043		0.189	
CES													
rs146390073^b^	RGS7	1	T/C	0.022	211,763	0.669	0.120	2.20 × 10^−8^	NA	NA		NA	
rs2466455	RP11-119H12.3	4	T/C	0.783	211,763	−0.299	0.022	2.75 × 10^−41^	0.018	0.037		0.626	
rs6838973	RP11-119H12.3	4	T/C	0.434	211,763	−0.108	0.020	3.58 × 10^−8^	−0.014	0.029		0.628	
rs12932445	ZFHX3	16	C/T	0.181	211,763	0.176	0.025	6.88 × 10^−13^	−0.017	0.044		0.696	

CES, cardio-embolic stroke; Ch, chromosome; E/O, effect/other; EAF, effect allele frequency; IS, ischemic stroke; LVS, large vessel atherosclerosis stroke; MR, mendelian randomization; NA, not applicable; SE, standard error; SNP, single nucleotide polymorphism.

a represented blood selenium level here.

b not included in the MR analysis.

**TABLE 3:** Sensitivity analysis of ischemic stroke and selenium levels.

	Pleiotropy		Heterogeneity	
	Intercept	*p*-value	Q	*p*-value
Exposures				
IS of all causes	0.124	0.404	4.426	0.352
LVS	−	−	4.887^a^	0.027
CES	0.027	0.672	0.157	0.692

IS, ischemic stroke; LVS, large vessel atherosclerosis stroke; CE, cardio-embolic stroke.

a by inverse variance weighted method.

## Discussion

By bidirectional MR analysis based on the summarized data of the GWAS, we found that neither selenium levels were causally associated with IS and its subtypes nor IS and its subtypes were causally associated with selenium levels. The results of our analysis were robust with multiple statistical methods, such as heterogeneity test, pleiotropy test, and leave-one-out analysis.

To our knowledge, the present study is the first study to investigate the causal links between selenium levels and IS and its subtypes by using the bidirectional MR method. Previously, the association between selenium levels and IS was controversial and not well investigated. Prior studies have revealed the potential protective role of selenium in cardiovascular disease. In a case–control study with more than 1,000 Chinese subjects, lower concentrations of selenium were associated with a higher risk of IS (Wen et al., 2019). The inverse association between selenium levels and prevalence of IS was also observed in American subjects (Hu et al., 2019). Nevertheless, Wu et al. (2021) revealed no association between baseline serum selenium levels and stroke in a cohort study (Wei et al., 2004). In a meta-analysis including 12 observational studies, circulating selenium levels were inversely associated with the risk of stroke (Ding and Zhang, 2021). However, in a subgroup analysis, the negative association of selenium levels and stroke was confirmed in the retrospective study group, but not in the prospective study group (Ding and Zhang, 2021). Therefore, the association between selenium levels and IS was controversial and not well investigated. Studies which demonstrated the association between selenium levels and IS with different etiologies were rare. Mironczuk et al. (2021) reported a higher copper-to-selenium ratio in CES patients but a relatively low copper-to-selenium ratio in SVS patients.

The association between selenium levels and stroke is complicated. Selenium is an essential trace element of the human body and shows antioxidant activity by scavenging free radicals (Fang et al., 2002). In the rodent IS model, pretreatment of selenium had significant protective effects on the activity of catalase, superoxide dismutase, and glutathione peroxidase (Ansari et al., 2004). In addition, selenium pretreatment significantly improved hypoxia/ischemia-induced neuron death and reduced infarction volume by alleviating oxidative stress and maintaining mitochondrial function (Mehta et al., 2012). However, the beneficial effect of selenium could be attenuated or even eliminated because of the increasing inflammation and oxidative stress caused by stroke (Ding and Zhang, 2021). Moreover, excess blood selenium concentration (130–150 μg/L) might be associated with minimal mortality (Rayman, 2012).

Gender differences could be a reason for the null finding. Hu et al. (2021) reported a negative association between selenium levels and the first stroke in males but not in females. Different sources (plasma, whole blood, diet, and environment) of selenium used in different studies could be another reason for the null finding and the discrepancy between the present and previous studies (Hu et al., 2017; Merrill et al., 2017; Hu et al., 2019; Wen et al., 2019; Xiao et al., 2019; Hu et al., 2021). Then, regarding the effect of IS on selenium levels, lower selenium levels were observed among acute IS patients in a retrospective study (Angelova et al., 2008). But our analysis provided no evidence of causal effects of IS on selenium levels. Wu et al. (2021) reported genetically predicted selenium levels were negatively causally associated with total cholesterol and low-density lipoprotein cholesterol, which were risk factors for IS (Diener and Hankey, 2020). Furthermore, selenium was reported to be positively correlated with systemic arterial function (Chan et al., 2012). Because previous studies reported non-linear association (including J-shaped and U-shaped) between selenium levels and stroke, the links between selenium levels and IS are rather complicated and still need further investigation (Bleys et al., 2008; Hu et al., 2017; Hu et al., 2019; Hu et al., 2021).

Given the antioxidant activity of selenium and selenoproteins, selenium supplementation was proposed as a potential strategy for the prevention of multiple disorders, like IS, osteoarthritis, rheumatoid arthritis, hypothyroidism, and prostate cancer (Sanmartin et al., 2011). Regarding stroke, selenium supplementation directly into the brain induced the expression of antioxidant glutathione peroxidase 4, which further inhibited the ferroptosis of neurons in a brain hemorrhage model (Alim et al., 2019). In a clinical trial of 29,584 Chinese people, the group receiving selenium supplements for a period of 5 years had a reduction in stroke mortality (9%), but no statistical significance was identified (Mark et al., 1998). Through a secondary analysis of the Nutritional Prevention of Cancer Trial, Stranges et al demonstrated no beneficial effect of selenium supplementation on stroke or cardiovascular disease incidence (Stranges et al., 2006). By bidirectional MR analysis, our results did not support the effectiveness of selenium supplementation in the prevention of IS and its subtypes at the genetic level. Given the impact of selenium levels on blood lipids and arterial function (Chan et al., 2012; Wu et al., 2021), the efficacy of selenium supplementation in subjects with hyperlipidemia or atherosclerotic lesions needed further investigation.

There were some limitations to our study. First, only subjects with European ancestry were included in the MR analysis. The prevalence and incidence of IS vary with ethnicity and so do the proportions of the subtypes of IS (Kim and Kim, 2014). Studies of Western populations indicated CES was the most common subtype of IS, while studies in Asian countries reported a higher prevalence of LVS than CES (Kolominsky-Rabas et al., 2001; Tsai et al., 2013). And the ethnicity differences among the SNPs associated with selenium levels also exist (Supplementary Table S5). Therefore, the results of this study needed further validation in Asian or African people. Second, despite including the genetic variants significantly associated with selenium levels from the largest GWAS of selenium levels, only 4 SNPs were finally included in MR analysis. While the 4 SNPs explained approximately 5.9% of the variance of selenium levels and the *F*-statistic of each SNP was more than 10. Therefore, more genetic variants associated with selenium levels, both blood and toenail selenium levels, need to be identified in the future. Third, pleiotropy, which is inevitable in MR analysis, may overestimate the effect of the exposure on the outcome. To eliminate the impact of pleiotropy as much as possible, we sought to identify potential pleiotropic SNPs before the MR analysis. By PhenoScanner, we found one SNP significantly associated with homocysteine. In addition, we performed a pleiotropy test by MR-Egger intercept, and no pleiotropy was found in the present study. Fourth, regarding outcome datasets of selenium levels, only blood selenium levels were used in the MR analysis. So, the causal effects of IS and its subtypes on toenail selenium levels are still unclear. Last, although our analysis suggested no effectiveness of selenium supplementation for patients with IS at the genetic level, large randomized controlled trials are needed to investigate the efficacy and safety of selenium supplementation for IS patients.

## Conclusion

In conclusion, our bidirectional MR study provides no evidence to support the causal links between genetically predicted selenium levels and IS. Our results also did not support the use of selenium supplementation for IS prevention at the genetic level. Clinical trials with high quality and large sample size are warranted to further elucidate the underlying association between selenium levels and IS and the clinical benefit of selenium supplementation for the prevention of IS.

## Data Availability

The original contributions presented in the study are included in the article/Supplementary Material, further inquiries can be directed to the corresponding author.

## Supplementary Material

Supplementary Figure S1 Sensitivity analysis of the causal effects of selenium levels on ischemic stroke. **(A)** Leave-one-out analysis of the causal effects of selenium levels on ischemic stroke and its subtypes. **(B)** Scatter plot analysis of the causal effects of selenium levels on ischemic stroke and its subtypes. **(C)** Forest plot analysis of the causal effects of selenium levels on ischemic stroke and its subtypes. **(D)** Funnel plot analysis of the causal effects of selenium levels on ischemic stroke and its subtypes. IS, ischemic stroke; LVS, large-vessel atherosclerosis stroke; CES, cardio-embolic stroke; SVS, small-vessel occlusion stroke; MR, Mendelian randomization; SNP, single-nucleotide polymorphism.

Supplementary Figure S2 Sensitivity analysis of the causal effects of ischemic stroke on blood selenium levels. **(A)** Leave-one-out analysis of the causal effects of ischemic stroke and its subtypes on blood selenium levels. **(B)** Scatter plot analysis of the causal effects of ischemic stroke and its subtypes on blood selenium levels. **(C)** Forest plot analysis of the causal effects of ischemic stroke on blood selenium levels. **(D)** Funnel plot analysis of the causal effects of ischemic stroke on blood selenium levels. IS, ischemic stroke; LVS, large-vessel atherosclerosis stroke; CES, cardio-embolic stroke; SVS, small-vessel occlusion stroke; MR, Mendelian randomization; SNP, single-nucleotide polymorphism.
