# Supplementary material for: A Bidirectional Mendelian Randomization Study of Selenium Levels and Ischemic Stroke
Source: Front Genet. 2022 Apr 13;13:782691. doi: 10.3389/fgene.2022.782691 (PMC9043360; doi:10.3389/fgene.2022.782691)
Supplement: Supplementary file 1 [file Table1.docx]

**Supplementary Table 1. Power calculation for bidirectional Mendelian randomization analysis of selenium levels and ischemic stroke.**

| Exposure | Outcome | Sample size | *K* | *R*^2^ | OR |
| --- | --- | --- | --- | --- | --- |
| Se | IS | 440,328 | 0.084 | 0.059 | 0.94/1.06 |
| Se | LVS | 150,765 | 0.030 | 0.059 | 0.85/1.18 |
| Se | CES | 211,763 | 0.035 | 0.059 | 0.88/1.14 |
| Se | SVS | 198,048 | 0.028 | 0.059 | 0.86/1.16 |
| IS | Se | 9,639 | 0.084 | 0.012 | 0.50/1.99 |
| LVS | Se | 9,639 | 0.030 | 0.025 | 0.48/2.07 |
| CES | Se | 9,639 | 0.035 | 0.045 | 0.57/1.74 |

CES: cardio-embolic stroke; IS: ischemic stroke; *K*: proportion of cases in the study; LVS: large vessel atherosclerosis stroke; OR: odds ratio; *R*^2^: the proportion of variance explained by the instrumental variables; Se: selenium; SVS: small vessel occlusion stroke.
