# Supplementary figures and images for: A Bidirectional Mendelian Randomization Study of Selenium Levels and Ischemic Stroke
Source: Front Genet. 2022 Apr 13;13:782691. doi: 10.3389/fgene.2022.782691 (PMC9043360; doi:10.3389/fgene.2022.782691)

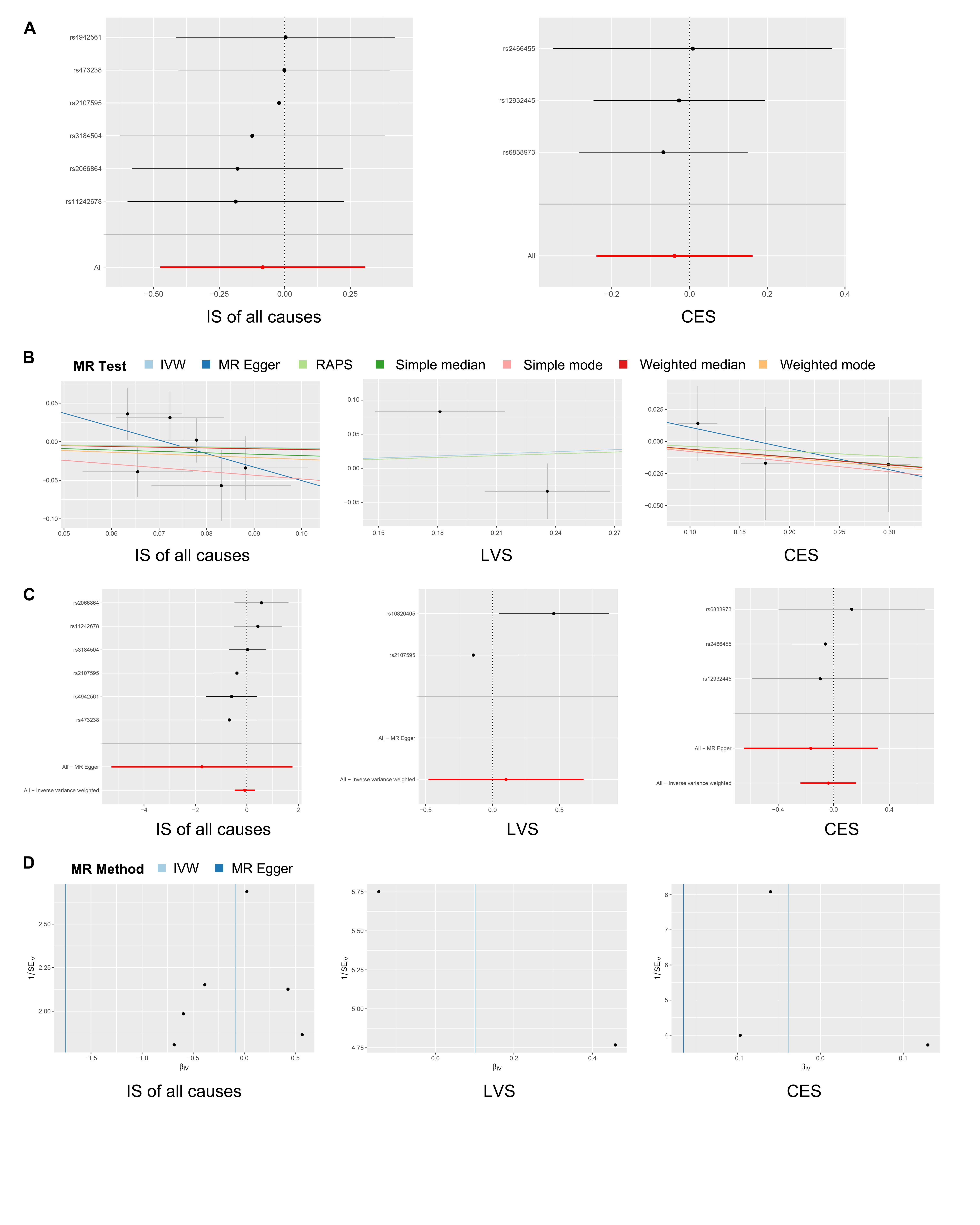

Supplement: Supplementary file 2 [file Image2.tif]

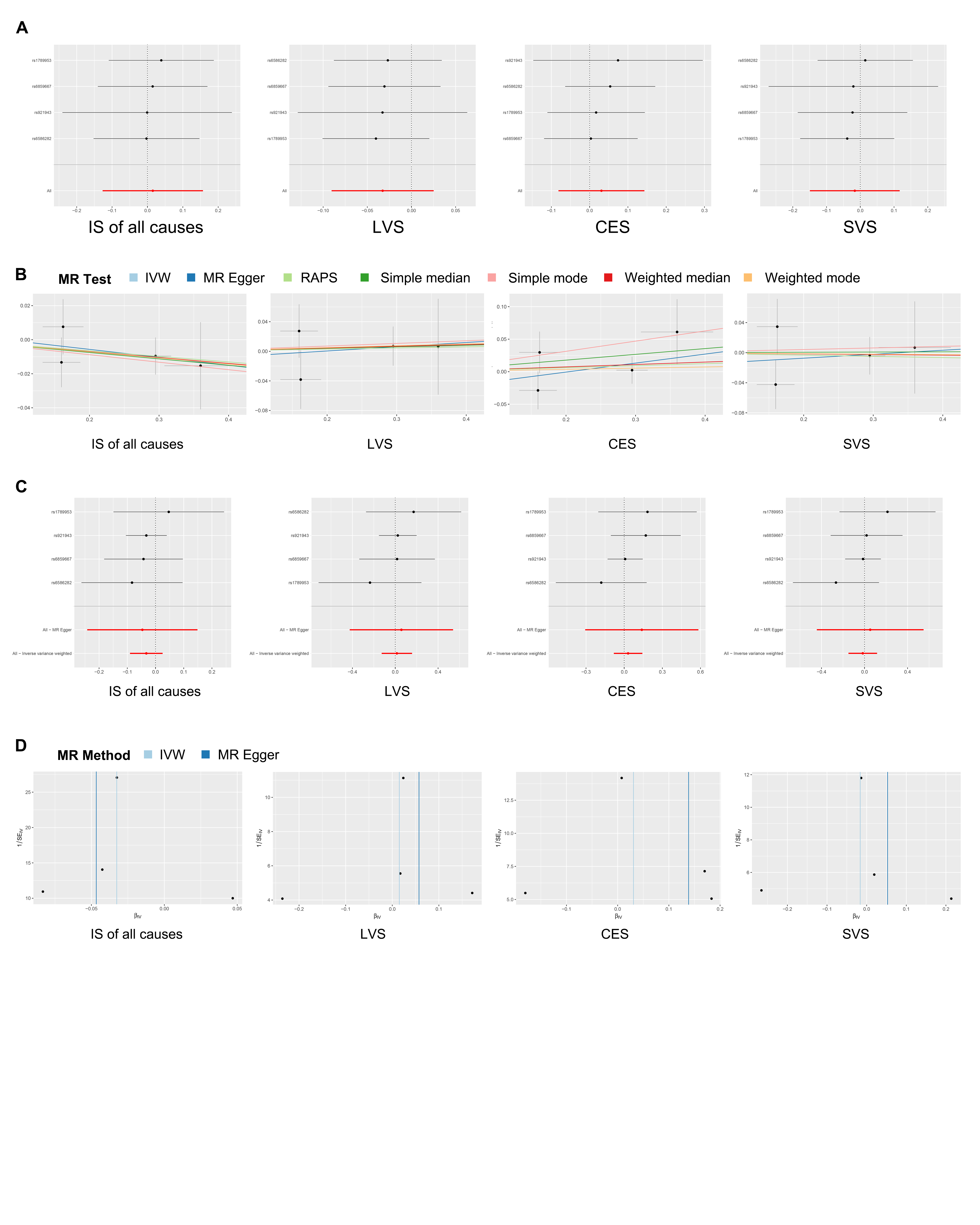

Supplement: Supplementary file 3 [file Image1.tif]
