# Supplementary material for: A Bidirectional Mendelian Randomization Study of Selenium Levels and Ischemic Stroke
Source: Front Genet. 2022 Apr 13;13:782691. doi: 10.3389/fgene.2022.782691 (PMC9043360; doi:10.3389/fgene.2022.782691)
Supplement: Supplementary file 4 [file Table2.docx]

**Supplementary Table 2. MR results of the effect of selenium levels on ischemic stroke.**

| SNP | IS of all causes | |  | LVS | |  | CES | |  | SVS | |
| --- | --- | --- | --- | --- | --- | --- | --- | --- | --- | --- | --- |
|  | OR (95% CI) | *p* |  | OR (95% CI) | *p* |  | OR (95% CI) | *p* |  | OR (95% CI) | *p* |
| rs921943 | 0.99 (0.97-1.01) | 0.379 |  | 1.01 (0.96-1.06) | 0.791 |  | 1.00 (0.96-1.04) | 0.911 |  | 1.00 (0.95-1.05) | 0.870 |
| rs6859667 | 0.98 (0.94-1.04) | 0.552 |  | 1.01 (0.89-1.14) | 0.924 |  | 1.06 (0.96-1.17) | 0.226 |  | 1.01 (0.89-1.14) | 0.911 |
| rs6586282 | 1.01 (0.98-1.04) | 0.362 |  | 0.97 (0.91-1.04) | 0.449 |  | 1.03 (0.97-1.09) | 0.323 |  | 1.04 (0.98-1.11) | 0.194 |
| rs1789953 | 1.01 (0.98-1.04) | 0.638 |  | 0.96 (0.89-1.04) | 0.336 |  | 1.03 (0.97-1.10) | 0.352 |  | 1.04 (0.96-1.11) | 0.348 |

MR: mendelian randomization; SNP: single nucleotide polymorphism; IS: ischemic stroke; LAS: large vessel atherosclerosis stroke; CE: cardio-embolic stroke; SVS: small vessel occlusion stroke; OR: odds ratio; CI: confidential interval.
