# Supplementary material for: A Bidirectional Mendelian Randomization Study of Selenium Levels and Ischemic Stroke
Source: Front Genet. 2022 Apr 13;13:782691. doi: 10.3389/fgene.2022.782691 (PMC9043360; doi:10.3389/fgene.2022.782691)
Supplement: Supplementary file 5 [file Table5.docx]

**Supplementary Table 5. Detailed information about the SNPs significantly associated with selenium levels among different populations.**

| SNP | MAF | | |
| --- | --- | --- | --- |
|  | European | Asian | African |
| rs921943 | 0.29 | 0.17 | 0.47 |
| rs6859667 | 0.04 | <0.01 | 0.07 |
| rs6586282 | 0.17 | 0.00 | 0.24 |
| rs1789953 | 0.14 | 0.17 | 0.09 |

SNP: single nucleotide polymorphism; MR: mendelian randomization; Ch: Chromosome; SE: standardized error; E/O: Effect or other; MAF: minor allele frequency.
