# Supplementary material for: A Bidirectional Mendelian Randomization Study of Selenium Levels and Ischemic Stroke
Source: Front Genet. 2022 Apr 13;13:782691. doi: 10.3389/fgene.2022.782691 (PMC9043360; doi:10.3389/fgene.2022.782691)
Supplement: Supplementary file 6 [file Table3.docx]

**Supplementary Table 3. Sensitivity analysis of selenium levels and ischemic stroke**

|  | Pleiotropy | |  | Heterogeneity | |
| --- | --- | --- | --- | --- | --- |
|  | Intercept | *p* |  | Q | *p* |
| Outcomes | | | | | |
| IS of all causes | 0.004 | 0.896 |  | 0.937 | 0.626 |
| LVS | -0.011 | 0.874 |  | 1.502 | 0.472 |
| CES | -0.028 | 0.669 |  | 2.699 | 0.259 |
| SVS | -0.018 | 0.802 |  | 2.453 | 0.293 |

IS: ischemic stroke; LVS: large vessel atherosclerosis stroke; CES: cardio-embolic stroke; SVS: small vessel occlusion stroke.
