# Supplementary material for: A Bidirectional Mendelian Randomization Study of Selenium Levels and Ischemic Stroke
Source: Front Genet. 2022 Apr 13;13:782691. doi: 10.3389/fgene.2022.782691 (PMC9043360; doi:10.3389/fgene.2022.782691)
Supplement: Supplementary file 7 [file Table4.docx]

**Supplementary Table 4. Sensitivity analysis of ischemic stroke and selenium levels.**

|  | Pleiotropy | |  | Heterogeneity | |
| --- | --- | --- | --- | --- | --- |
|  | Intercept | *p*-value |  | Q | *p*-value |
| Exposures | | | | | |
| IS of all causes | 0.124 | 0.404 |  | 4.426 | 0.352 |
| LVS | - | - |  | 4.887^*^ | 0.027 |
| CES | 0.027 | 0.672 |  | 0.157 | 0.692 |

IS: ischemic stroke; LVS: large vessel atherosclerosis stroke; CE: cardio-embolic stroke.

^*^: by inverse variance weighted method.
